# Online assessment of the perception of loneliness and associated factors in Colombian climacteric women during the COVID-19 pandemic: A cross-sectional study

**DOI:** 10.34172/hpp.2021.28

**Published:** 2021-05-19

**Authors:** Angélica Monterrosa-Blanco, Álvaro Monterrosa-Castro, Andrea González-Sequeda

**Affiliations:** ^1^Health Sciences University Foundation (FUCS), Women’s Health Research Group, Faculty of Medicine, University of Cartagena, Cartagena, Colombia; ^2^Research Department, Women’s Health Research Group. Faculty of Medicine, University of Cartagena, Cartagena, Colombia; ^3^Women’s Health Research Group, Faculty of Medicine, University of Cartagena, Cartagena, Colombia

**Keywords:** Anxiety, Climacteric, COVID-19, Fear, Loneliness, Mental health, Middle aged, Pandemics, Quality of life

## Abstract

**Background:** The coronavirus disease 2019 (COVID-19) pandemic has generated changes due to confinement, this measure can increase the perception of loneliness. The objective was to estimate the frequencies of emotional, social and general loneliness and their association with fear and anxiety with COVID-19, religiosity and severe deterioration of quality of life in middle-aged women.

**Methods:** A cross-sectional study in Colombian women (40-59 y, n=984) surveyed with an electronic form that included sociodemographic characteristics and validated measures (Menopause Rating Scale, de Jong Gierveld Loneliness Scale, fear of COVID-19 scale, Coronavirus Anxiety Scale and Francis Scale for Religiosity). Associations of emotional, social and general loneliness (dependent variables) with severe somatic, psychological, urogenital and quality of life deterioration, as well as with high religiosity, anxiety and high fear of COVID-19 (independent variables), were estimated.

**Results:** The median age was 47 years old, and 39.2% [95% CI: 36.2-42.3] postmenopausal. Severe deterioration in somatic, psychological, urogenital domains and quality of life in women with emotional, social and general loneliness was found (*P* <0.001). In adjusted models, high fear of COVID-19, severe deterioration of psychological and urogenital domains and quality of life were associated with emotional, social and general loneliness. Anxiety with COVID-19, somatic domain and high religiosity were not associated with loneliness.

**Conclusion:** Emotional, social and general loneliness were identified in 4/10 middle-aged Colombian women surveyed, and the associated factors were high fear of COVID-19, severe deterioration of quality of life and psychological and urogenital domains. Professionals who care for climacteric women should explore the perception of loneliness when assessing menopausal symptoms.

## Introduction

On December 31, 2019, a new viral pneumonia originating from Wuhan, China, was issued by the World Health Organization (WHO).^1^ Since March 2020, severe acute respiratory syndrome coronavirus 2 (SARS-CoV-2) has spread throughout the world, and on May 2nd 2021, more than 152 million detected cases of coronavirus disease 2019 (COVID-19) and 3 196 298 deaths worldwide were indicated by Johns Hopkins University.^2^

Customs and daily life changed on all continents. Most governments imposed social distancing, quarantine or confinement to control the disease.^3-5^ Humans are social beings who experience intense suffering when a reduction in social connection is perceived.^6^ The general population, and therefore middle-aged women, were subjected to complex situations. COVID-19 is accompanied by fear, uncertainty, economic tension and other stressors, which are detrimental to mental health.^7-11^

One of the most common event derived from confinement is the perception of loneliness, defined as the discrepancy between the desired social relationships and those that exist in reality.^12-16^ A differentiation should be made between “feeling lonely” and “being alone”. The first refers to emotional loneliness, a feeling derived from the loss or absence of loved ones; it refers to subjective experiences that are not determined by the number of social contacts. Being alone refers to social isolation, the lack of company networks, marginalization, uprooting and the objective lack of social contacts.^17-19^

The perception of loneliness has been especially evaluated in older adults, which can generate the false assumption that it is an old age event.^19-21^ Although feeling lonely is a common complaint in the menopausal transition, it is not usually evaluated in relation to quality of life and menopausal symptoms.^12^ The available information in the Latin American population is insufficient to define the frequency of perceived loneliness in climacteric women and its association with psychosocial alterations or with the biological deterioration related to menopausal symptoms. It is worth studying fear, anxiety and religiosity in relation to the perception of loneliness in climacteric women during a pandemic. The aim of this study was to determine the frequencies of emotional, social and general loneliness and their association with fear and anxiety with COVID-19, religiosity and severe deterioration of the quality of life in climacteric middle-aged women.

## Materials and Methods

### Participants

This cross-sectional study was part of the CAVIMEC+COVID STUDY (quality of life in the menopausal and Colombian ethnicities under pandemic conditions). The research project included nonpregnant healthy women (40-59 years old) living in Colombia. A call was made through social networks (WhatsApp, Facebook and Instagram) and emails. Women residing in Colombia participated between June 1 and 5, 2020 by filling out an electronic form. Participants were asked to apply their responses according to their perceptions between May 1 and May 30, 2020. In that period of time, as a result of COVID-19, confinements and curfews were decreed by the national government in some Colombian cities. In addition, infection and death curves were rising daily.

Women were informed of the anonymous, confidential and voluntary nature of their participation, the research aims and the tools to be used, and they were requested to give their informed consent for participation. No incentives (e.g., money) were offered in exchange. The only exclusion criterion was leaving the form incomplete. The Declaration of Helsinki for medical research involving humans was considered, as well as the ethical principles of the Belmont Report. The research project has the institutional endorsement of the Universidad de Cartagena, Colombia and was approved by the Research Ethics Committee of Clínica Santa Cruz de Bocagrande, Cartagena, Colombia, according to act 03-2020 of March 21, 2020.

### Measures

An electronic form developed in Google Forms was applied. Google Forms is a Google Drive application used to conduct surveys and acquire statistics based on opinion. The form could be completed by accessing a link. It included sociodemographic variables: age, ethnicity, menstrual episodes, and number of children. Ethnicity was determined by self-recognition. Menopausal status was defined according to menstrual bleeding: those with current bleeding or bleeding that had been absent less than a year were classified as premenopausal, and those with amenorrhea of more than a year were classified as postmenopausal.

The form had five scales. First, the de Jong Gierveld Loneliness Scale - Short Version (DJGLS), consists of eleven items with three response categories: No, More or less and Yes. One point is awarded if the answer is “More or less” or “Yes” and none if the answer is “No” in items 2, 3, 5, 6, 9, and 10 (formulated negatively). In items 1, 4, 7, 8, and 11 (formulated positively), one point is assigned if the answer is “More or less” or “No” and none if the answer is “Yes”. The total score ranges from 0 (no loneliness) to 11 (extreme loneliness). Those items formulated negatively measure emotional loneliness, the others social loneliness, and all of them assess general loneliness. The scale can be used two-dimensionally to explore emotional and social loneliness or one-dimensionally to identify general loneliness. In the last decade, it has probably been the most widely used scale to measure loneliness and has been validated in several languages.^22-25^ For this study, emotional, social and general loneliness were considered with an above-average score of the surveyed women. There is no proposed cutoff point for the DJGLS. For this sample, a Kuder-Richardson coefficient of 0.87 was estimated for one-dimensional DJGLS, 0.81 for DJGLS-emotional loneliness and 0.79 for DJGLS-social loneliness.

Second, the Menopause Rating Scale (MRS), which is a specific quality of life scale in menopause, consists of eleven questions grouped into three domains: somatic, psychological, and urogenital. Quality of life is determined by the sum of the scores of the three domains. The higher the score, the worse the assessment of symptoms, domains and quality of life. Scores of >8 in somatic, >6 in psychological, > 3 in urogenital, and >16 in quality of life are defined as severe deteriorations of the respective domains. The MRS has been widely used and validated in different languages.^26,27^ For this group of women, Cronbach’s alpha value of 0.81 was found for the MRS.

Third, the fear of COVID-19 scale five-item version (FCV-19S-5) was validated in Spanish and in the Colombian population from its original version to assess fear of COVID-19. It has dichotomous response options and has a Kuder-Richardson coefficient of 0.67 and a McDonald omega of 0.68.^28^ For the study, an above-average score defined high fear of COVID-19, no cutoff point has been proposed. A Kuder-Richardson coefficient of 0.78 was estimated for fear of COVID-19 in this sample of women.

Fourth, the Coronavirus Anxiety Scale (CAS) was proposed in 2020 to assess five anxiety situations regarding the coronavirus, each rated from 0 to 4 points. A CAS score >9 optimally classified adults as having (90% sensitivity) or not having (85% specificity) dysfunctional levels of anxiety.^29^ There is no validation for the Spanish language. Items were translated from English to Spanish and then translated back (reverse translation) following the Task Force for Translation and Cultural Adaptation guidelines.^30^ Cronbach’s alpha value of 0.82 was found for the anxiety with COVID-19 scale in this group of women.

Fifth, the short form of the Francis scale explores attitudes towards Christianity in relation to God, Jesus and prayer. Five items with response options ranging from strongly disagree to strongly agree are scored from 0 to 4 points. In the Colombian population, Cronbach’s α of 0.74 has been reported.^31^ For this study, an above-average score was considered high religiosity, because there is no proposed cutoff point. Cronbach’s alpha value of 0.96 was estimated for the Francis scale in this group of women.

### Sample size

Sample size calculation was performed with data from the Colombian population census of 2005 that established a projection of 25 772 783 women for 2020; of these, 2,859,309 were aged 40 to 59 years old. A sample size of 664 women was calculated in the Epidemiological Analysis from Tabulated Data 3.1 (EPIDAT) software with a 99% confidence level, 50% expected proportion, 1% significance and 5% absolute precision.

### Statistical analysis

The database that was automatically generated in Microsoft Excel© was downloaded from the Google platform

(https://docs.google.com/forms/d/e/1FAIpQLSeh_as86kzZp6Tu1K2gFgZlpITumfO7j8jDGyop9GkWiVNbMg/viewform?vc=0&c=0&w=1&gxids=7628). Data were refined, participants’ emails were removed to preserve anonymity, and incomplete forms were discarded. The statistical analysis was performed with Stata IC version 16 (https://www.stata.com/new-in-stata/). Continuous data are expressed as medians and interquartile ranges (IQRs) because data distribution was non parametric, normality was evaluated with graphical methods (histogram and kernel density) and numerical methods (skewness/kurtosis and Shapiro-wilk). Categorical data are expressed in absolute values and percentages. The differences between groups were evaluated with the Student’s *t* test or Mann-Whitney U (according to the homogeneity of the variance for continuous variables) and chi-square or Fisher’s exact test for categorical variables, according to the expected values. Bivariate analysis was performed to establish an association between emotional, social and general loneliness with severe somatic, psychological, urogenital and quality of life deterioration, high fear of COVID-19, dysfunctional levels of anxiety with COVID-19 and high religiosity. It was preferred to associate loneliness with the severe deterioration of the MRS domains and not with the presence or absence of just deterioration, due to the clinical implications that underlie alterations of greater severity. Six multivariate logistic regression were performed to estimate adjusted OR, the three first models consider emotional, social and general loneliness as dependent variables, respectively. The three of them included the independent variables: severe somatic deterioration, severe psychological deterioration, severe urogenital deterioration, high fear of COVID-19, dysfunctional levels of anxiety with COVID-19 and high religiosity.

On the other hand, the fourth, fifth and sixth models also consider emotional, social and general loneliness as dependent variables, respectively. Instead, the independent variables were: severe deterioration of quality of life, high fear of COVID-19, dysfunctional levels of anxiety with COVID-19, high religiosity, age range and menopausal status. These variables were included with the intention to identify loneliness behavior when chronological change and menstrual condition are considered. The goodness of fit of each model was estimated with the Hosmer-Lemeshow test (*P*>0.05). Additionally, the Akaike’s information criterion and Bayesian information criterion were identified to estimate the most appropriated model. Internal consistency was estimated in this population for the five scales included in the study. A *P* < 0.05 was considered statistically significant.

## Results

At the first five days of June 2020, 1012 forms were received, 28 (2.7%) had incomplete data, thus were discarded. A total of 984 women were included in the analysis, 320 (48.2%) above the calculated size. The median age of the total sample was 48.0 years old (IQR: 42.0–53.5). A total of 84.5% of surveyed women were Hispanic, 13.7% were Afro-descendant, and 1.7% were indigenous; 39.2% were postmenopausal.

The average score in the surveyed women for emotional loneliness was 2.48 ± 2.09, for social loneliness 2.14 ± 1.80 and for general loneliness 4.62 ± 3.52. The previous values are considered the cut-off points in the evaluated group. Emotional loneliness was identified in 433 participants (44.0%, 95% confidence interval (95% CI): 40.9–47.1), social loneliness in 415 participants (42.2%, 95% CI: 39.1–45.3) and general loneliness in 438 participants (44.5%, 95% CI: 41.4–47.6). Greater levels of emotional, social and general loneliness were noted in women between 40 and 44 years old than in other age groups. In more than half of the evaluated women, high fear of COVID-19 and high religiosity were found, while in 7.9%, dysfunctional levels of anxiety with COVID-19 were identified. Table 1 presents the sociodemographic characteristics.

More than 60% answered affirmatively three of the DJGLS items: “I can call on my friends whenever I need”, “there are plenty of people I can lean on when I have problems” and “there is always someone I can talk to about my day-to-day problems” (Table 2). Among the women who presented emotional, social and general loneliness, greater frequencies of severe somatic, psychological, urogenital and quality of life deterioration were found (*P* < 0.001) (Table 3).

In the bivariate logistic regression, severe somatic, psychological, urogenital, and quality of life deterioration, high fear of COVID-19, dysfunctional levels of anxiety with COVID-19 and high religiosity were associated with emotional loneliness. All of these variables, except high religiosity, were also significantly associated with social and general loneliness (Table 4).

In the three first adjusted logistic regression models, high fear of COVID-19 was associated with 1.39, 95% CI (1.05-1.84); 1.54 (1.16-2.03) and 1.48, (1.12-1.96) -fold higher emotional, social and general loneliness, respectively (*P* < 0.05), in presence of the other variables. Similar figures were found with severe psychological and urogenital deterioration. Anxiety with COVID-19 and severe somatic deterioration lost statistical significance in the association with the three types of loneliness in presence of the other variables. Religiosity was not an associated factor with loneliness (Table 5). The goodness of fit with the Hosmer-Lemeshow test for the first three models was: 0.77, 0.92 and 0.95, respectively.

In the other three models, severe deterioration of quality of life was associated with emotional, social and general loneliness OR: 3.96, 95% CI (2.69-5.84), OR: 2.37 (1.64-3.41) and OR: 2.93 (2.01-4.27), respectively, in presence of the variables fear of COVID-19, dysfunctional levels of anxiety with COVID-19, religiosity, age range and menopausal status. The goodness of fit with the Hosmer-Lemeshow test for the last three models was: 0.97, 0.67 and 0.92, respectively.

The most appropriated model identified through the Akaike’s information criterion and Bayesian information criterion, was the first one, which considered as dependent variable emotional loneliness and as independent variables: severe somatic deterioration, severe psychological deterioration, severe urogenital deterioration, high fear of COVID-19, dysfunctional levels of anxiety with COVID-19 and high religiosity.

## Discussion

In the 984 women included in the study, greater frequencies of emotional, social and general loneliness were found in those between 40 and 44 years. Women with loneliness had greater severe somatic, psychological, urogenital and quality of life deterioration. In the bivariate analysis, severe somatic, psychological, urogenital and quality of life deterioration, high fear of COVID-19 and dysfunctional levels of anxiety with COVID-19 were associated with emotional, social and general loneliness. In the three first adjusted logistic regressions, high fear of COVID-19, severe psychological and urogenital deterioration continued to be associated with the three types of loneliness identified by the DJGLS. By involving menopausal status and age range in the last three regression models, only severe deterioration of quality of life was found to be significantly associated with emotional, social and general loneliness. Religiosity was not associated with loneliness.

Perception of loneliness is defined as an individual and subjective experience which is highly prevalent in old age and in people with emotional disorders; however, it also affects younger people.^17,19,32,33^ It has been related with mental health deterioration, low self-esteem, sleep disorders, depression, suicide attempts, poor quality of life, cardiovascular morbidity and increases the risk for all-cause mortality.^12,16,17,20,21,34^ Weiss^33^ determined that emotional loneliness is the loss or absence of a close person, a generator of emotional attachment. While social loneliness refers to the absence of an attractive social network or a wide circle of friends. Emotional loneliness is more common than social loneliness and is more related to loss of health.^16,34^

In this study, during the COVID-19 pandemic, four out of ten women in the vital climacteric stage reported emotional (44.0%), social (42.2%) and general (44.5%) loneliness. In a study of psychological well-being in relation to COVID-19 in the initial phase of confinement, in a population from 18 to 87 years old, investigators found a 27% prevalence of loneliness.^35^ In April 2020, in the same pandemic, 13% of American adults reported feeling lonely frequently, compared with a 2018 national survey that found an 11% prevalence of feelings of loneliness.^8^ In Switzerland, a study conducted in adults over 65 years old to determine the impact of COVID-19 on loneliness, pointed out that female sex, not having children, living alone, having low economic income and being dissatisfied with neighbors can be predictors for the appearance of loneliness.^7^ Cultural factors and differences in living conditions according to countries are important for explaining the differences in the prevalence of loneliness. This trend tends to be higher in central and southern Europe than in northern Europe; at the same time, there are differences between Western and Eastern Europe.^36-38^ Sundström et al^39^ in a study in twelve countries, found that Switzerland, Denmark and Sweden presented the lowest loneliness rates, while the highest loneliness rates were in France, Israel, Italy and Greece. Furthermore, the frequency of “feeling lonely most of the time” ranged from 1% in Switzerland to 7% in Spain and 10% in Greece.

In the Latin American social organization, and especially in Colombia, great importance is given to the concept of familism, a Latin cultural construct understood as the social attitude towards family values, the prioritization of family, the need for family members to provide and receive support and to be ready to defend the family institution.^40^ The social distancing and quarantine established by authorities due to the COVID-19 pandemic broke family ties and violated the family environment interests. This may explain the high frequency of the three types of loneliness that were found in middle-aged women. It is possible to speculate that in the cultural context of Amerindian communities, the perception of loneliness is greater. This may be influenced by the following circumstances: distancing from family members, impediments to community coexistence, limits to interrelationships with the neighborhood, lack of social support and restrictions on friendship relationships.^16,19,41^ Chiao et al^42^ pointed out that young adults with strong family cohesion in their adolescence are less likely to suffer from severe emotional loneliness (relative risk ratio (RRR) : 0.77 [95% CI: 0.65–0.91]). They also indicate that loneliness patterns are associated with family characteristics.

Several authors^17,43,44^ describe that in addition to the social network size, the quality of personal interrelationships affects feelings of loneliness and the effect generated by distancing and social isolation. A meta-analysis by Pinquart and Sörensen^45^ suggests that the quality of social relationships has an explanatory power for loneliness that is three times higher than that of the number of social contacts. In the same way, Hawkley and Cacioppo^46^ state that it is better to have social relationships that offer security, comfort, social support, trust and pleasure, even if contact is infrequent, than to have fewer intimate friends with frequent interactions. An adequate social support network that offers the exchange of resources, goods, services and affections reduces the perception of loneliness. Although social support and loneliness are two different constructs, both are negatively related to each other.^7,17,19^

It was found that younger women, compared with older women, had a greater perception of loneliness. The same is true when comparing premenopausal women with postmenopausal. Losada-Baltar et al^13^ report similar findings in the Spanish population in both loneliness, sadness and anxiety. They indicate that age is a risk factor for experiencing mental health problems associated with quarantine due to sanitary conditions such as those generated by COVID-19. The younger the age is, the greater the risk of negative emotional symptoms. The foregoing can be explained since the youngest were more socially active before the pandemic. Groarke et al^35^ found that belonging to the youngest age group was a risk factor for loneliness in the English population. Feelings of loneliness can arise when social isolation is imposed from the outside, forcing people to live alone or to be alone against their desires.^15,17,21^ Social distancing should not be confused with social isolation, particularly not today, when connectivity and virtuality can generate rapprochements, contributing to the prevention of both emotional and social loneliness.^13^

In the vital stage of the climacteric, the different health interventions should seek the conservation of quality of life and the prevention of biopsychosocial deterioration.^12,47^ We identified that severe psychological deterioration was associated more than twice with the perception of emotional, social and general loneliness. Regardless of whether loneliness is explored from a one-dimensional (general) or two-dimensional (emotional and social) perspective, it is possible to determine its relationship with the deterioration of the psychological sphere.^16,17,19,20^ Adults with perceptions of loneliness will have greater difficulties regulating their emotions, with a predominance of negative thoughts and mood over positive ones. This behavior, and the fact of being alone or feeling lonely, can increase in times of crisis generated by epidemics, in which the spaces for face-to-face interaction with either family or friends are limited.^13^

In Spanish women aged between 40 and 63 years old, the UCLA Loneliness Scale scores were positively correlated with the psychological dimension of the MRS scale, with alcohol abuse within the couple relationship and with living in urban areas, while an inverse correlation was noted with life satisfaction and stability of the couple relationship.^12^ In this study, women with emotional, social and general loneliness had greater severe psychological, urogenital and quality of life deterioration. Hombrados-Mendieta et al^48^ found that frequency and satisfaction with couple support were negatively related to the perception of loneliness. It is essential that women in the climacteric stage strengthen their interpersonal relationships and the development of their social life, even with the current virtual tools.^47^ Emotional support is accompanied by the greatest influence on reducing loneliness.^48^ Although a favorable association between loneliness and religiosity was not observed in this investigation, other authors^49^ have established that in the elderly, the implementation of religious activity reduced the loneliness sensation and increased mental vitality.

The perception of loneliness has been associated with changes in different spheres of human development: physical, spiritual, social, cognitive and environmental.^15-17,50^ In this study, half of the studied women reported having high fear of COVID-19, an emotional condition that was significantly associated with the perception of the three types of loneliness explored in both the adjusted and unadjusted models. Increases of approximately two times in the unadjusted model and between 1.39 and 1.54 times in the adjusted model were identified. In the current COVID-19 pandemic, as has been noted in various epidemics, unwanted behaviors increased, especially fear, depression, anxiety and discrimination.^7,10,11,13,35^ Fear is a negative feeling that spreads faster than the epidemics and can be associated with other social conditions, such as stigmatization, discrimination, as well as mental health.^51^

Domènech-Abella et al^52^ in a prospective study in 5,066 Irish people over 50 years of age, found an association between loneliness and anxiety. We found similar data in this study; however, only in the unadjusted analysis, dysfunctional levels of anxiety with COVID-19 associated 3.5 times with emotional loneliness, 2.4 times with social loneliness and 2.6 times with general loneliness.

Although most evaluations of the perception of loneliness associate with a negative experience, some authors indicate that loneliness could be positive. According to existentialism, loneliness is positive when it results from one’s own choice to spend time alone and is characterized by an opportunity to reflect, communicate with God, and understand oneself.^17,19^ Although it was not investigated in this study, it will be important to explore the proportion of freely chosen loneliness in the different population conglomerates. Loneliness is a complex construct with biopsychosocial influences, it is experienced in different ways, permanently or temporarily; the latter is also called acute loneliness. Being alone or living alone is not the same as feeling alone.^7,15,17,19^ However, Abdellaoui et al^6^ affirmed that there is convincing evidence of a genetic predisposition to loneliness. Possessing a genetic predisposition towards loneliness was associated with cardiovascular, psychiatric, lipid and metabolic disorders.

The strength of this study is that it is one of the first to address the perception of loneliness in middle-aged women subjected to the pressures of a pandemic. Studies on the perception of loneliness in the climacteric stage are scarce; most are carried out in older adults. Others evaluate population groups with a wide age range and combine men with women. It is possible that this study is the first exploratory approach to the perception of loneliness and quality of life in Latin American climacteric women, who have their own sociocultural and ethnic influences. The tools used allow comparisons with data from other latitudes. Quality of life was determined with MRS, which has translations, applications, and validations in various languages. The DJGLS and Francis Scale are frequently used, while the FCV-19S-5 and CAS allow specific explorations of fear and anxiety related to COVID-19, respectively. The virtual recruitment and electronic collection of the information, are both strengths and limitations. It was possible to collect data quickly, at low cost and reach distant geographic regions; however, it was not possible to verify that participants adequately met the inclusion criteria.

This study has the inherent limitations of cross-sectional studies, and the results are statistical associations and do not indicate causality. No questions were asked about family or personal anxiety traits, nervous temperament, number of cohabiting people, sexual partners, outings to carry out work or other activities, noncompliance with confinement measures, influence of press news, comorbidities, use of medications and the perception of loneliness before the pandemic, all of which can generate information biases or confusion variables. Although a number of participants greater than the sample size was included, it can be considered a convenience sampling, therefore the results cannot be generalized to all the Colombian population. It is possible an overestimation or underestimation of the results, with possible measurement bias, given the impossibility of knowing the availability of access to connectivity of the social networks, by middle-aged women living in Colombia. Extensive studies are warranted in women in the vital stage of the climacteric, in different geographic and social contexts, in the midst and outside of a pandemic, to specify the relationships between loneliness, quality of life and different psychosocial factors, taking into account that there are traditional and cultural reasons that have important roles. Studies that better explore the relationship between loneliness and mental and physical health in middle-aged women are warranted.^6,16^

To reduce the spread of epidemics, government officials who impose social distancing measures are advised to enforce them wisely.^9,11^ This is especially true because of the consequences derived in terms of mental health and well-being, such as their association with the perception of loneliness.^3-5,17-19,53^

## Conclusion

Four out of ten middle-aged Colombian women surveyed had perceptions of emotional, social and general loneliness. High fear of COVID-19 and severe psychological and urogenital deterioration were associated factors. Professionals who care for climacteric women should routinely include questions or assessment tools of the perception of loneliness when assessing quality of life and menopausal symptoms. They should take into account that middle-aged women are exposed to hormonal adjustments related to menopause and to the influence of different social and emotional experiences.^12^ They should also be clear that the feeling of loneliness does not have signs or symptoms; it is subjective and can only be described individually according to internal experiences. Likewise, the loss of social relationships can have a negative impact on the meaning of life and even on how the person is perceived. The meaning of life is related to various values, especially self-esteem and self-efficacy. Loneliness is an example of the deficit of social relationships.^19^

## Acknowledgements

The authors thank the Universidad de Cartagena - Colombia, for the logistical and academic support. The authors are also deeply grateful to those who participated in the study.

## Funding

No funding was received to support this project.

## Competing interests

The authors declare that they have no competing interests.

## Ethical approval

This research was approved by the Research Ethics Committee of Clinica Santa Cruz de Bocagrande, Cartagena, Colombia, according to act 03-2020 of March 21, 2020. All the participant provided their informed consent.

## Authors’ contribution

AM-C, AM-B and AG-S participated in the study design and coordination and helped draft the manuscript. In addition, AM-B performed the statistical analysis. All authors wrote, read and approved the final manuscript.

**Table 1:** Sociodemographic characteristics of studied women

**Variables**	**Total** **984**	**Emotional Loneliness**			**Social Loneliness**			**General Loneliness**		
		**Yes**	**No**	***P***	**Yes**	**No**	***P***	**Yes**	**No**	***P***
		**433 (44.0%)**	**551 (56.0%)**		**415 (42.2%)**	**569 (57.8%)**		**438 (44.5%)**	**546 (55.5%)**	
Age, y, Me [IQR]	47.0 [42.0-53.5]	46.0 [41.0-54.0]	47.0 [41.0-54.0]	0.27^a^	46.0 [41.0-53.0]	48.0 [42.0-54.0]	0.008^a^	46.0 [41.0-53.0]	48.0 [42.0-59.0]	0.004^a^
Children, n, Me [IQR]	2.0 [1.0-2.0]	2.0 [1.0-2.0]	2.0 [1.0-2.0]	0.26^b^	2.0 [1.0-2.0]	2.0 [1.0-2.0]	0.76^a^	2.0 [1.0-2.0]	2.0 [1.0-2.0]	0.09^a^
Age at menopause onset (among postmenopausal), Me [IQR]	49.0 [47.0-51.0]	49. 0 [47.0-50.0]	49.0 [47.0-51.0]	0.63^a^	49.0 [47.0-51.0]	49.0 [47.0-51.0]	0.51^a^	49.0 [47.0-50.0]	49.0 [47.50]	0.66^a^
Years since last menstruation (among postmenopausal), Me [IQR]	5.0 [3.0-7.0]	5.0 [3.0-8.0]	4.0 [2.0-7.0]	0.05^a^	5.0 [2.0-7.0]	5.0 [3.0-7.0]	0.10^a^	5.0 [3.0-8.0]	5.0 [2.0-7.0]	0.16^a^
40-44, y, n (%)	392 (39.8)	180 (41,5)	212 (38.4)	0.32^c^	182 (43.8)	219 (36.9)	0.02^c^	196 (44.7)	196 (35.9)	0.005^c^
45-49, y, n (%)	189 (19.2)	79 (18.2)	110 (19.9)	0.49^c^	82 (19.7)	107 (18.8)	0.70^c^	80 (18.2)	109 (19,9)	0.50^c^
50-54, y, n (%)	211 (21.4)	89 (20.5)	122 (22.1)	0.54^c^	86 (20.7)	125 (21.9)	0.63^c^	87 (19.8)	124 (22.7)	0.27^c^
55-59, y, n (%)	192 (19.5)	85 (19.6)	107 (19.4)	0.93^c^	65 (15.6)	127 (22.3)	0.009^c^	75 (17.1)	117 (21.4)	0.09^c^
Premenopausal, n (%)	598 (60.7)	270 (62.3)	328 (59.5)	0.36^c^	279 (67.2)	319 (56.0)	<0.001^c^	293 (66.8)	305 (55.8)	<0.001^c^
Postmenopausal, n (%)	386 (39.2)	163 (37.6)	223 (40.4)		136 (32.7)	250 (43.9)		145 (33.1)	241 (44.1)	
Hispanics, n (%)	832 (84.5)	361 (83.3)	471 (85.4)	0.36^c^	336 (80.9)	496 (87.1)	0.008^c^	361 (82.4)	471 (86.2)	0.09^c^
Afro-descendant, n (%)	135 (13.7)	62 (14.3)	73 (13.2)	0.62^c^	70 (16.8)	65 (11.4)	0.01^c^	67 (15.3)	68 (12.4)	0.19^c^
Indigenous, n (%)	17 (1.7)	10 (2.3)	7 (1.2)	0.21^c^	9 (2.17)	8 (1.4)	0.36^c^	10 (2.2)	7 (1.2)	0.23^c^
High fear of COVID-19, n (%)	564 (57.3)	288 (66.5)	276 (50.0)	<0.001^c^	276 (66.5)	288 (50.6)	<0.001^c^	291 (66.4)	273 (50.0)	<0.001^c^
Dysfunctional levels of anxiety with COVID-19, n (%)	78 (7.9)	56 (12.9)	22 (3.9)	<0.001^c^	49 (11.8)	29 (5.1)	<0.001^c^	52 (11.8)	26 (4.7)	<0.001^c^
High religiosity, n (%)	604 (61.3)	285 (65.8)	319 (57.8)	0.01^c^	254 (61.2)	350 (61.5)	0.92^c^	280 (63.9)	324 (59.3)	0.14^c^

^a^ Student’s t-test, ^b^ Mann-Whitney, ^c^ Chi-square. Quantitative data are presented as medians (Me) with interquartile ranges (IQR) Categorical data are presented in frequencies n (%).

**Table 2:** De Jong Gierveld Loneliness Scale

	**Items**	**Yes** **No. (%)**	**More or less** **No. (%)**	**No** **No. (%)**
1	There is always someone I can talk to about my day-to-day problems^a^	628 (63.9)	285 (28.9)	71 (7.2)
2	I miss having a really close friend^b^	216 (21.9)	217 (22.1)	551 (56.0)
3	I experience a general sense of emptiness^b^	115 (11.7)	233 (23.7)	636 (64.6)
4	There are plenty of people I can lean on when I have problems^a^	630 (64.0)	279 (28.4)	75 (7.6)
5	I miss the pleasure of the company of others^b^	250 (25.4)	248 (25.3)	486 (49.3)
6	I find my circle of friends and acquaintances too limited^b^	259 (26.3)	198 (20.2)	527 (53.5)
7	There are many people I can trust completely^a^	391 (39.7)	337 (34.3)	256 (26.0)
8	There are enough people I feel close^a^	524 (53.2)	268 (27.3)	192 (19.5)
9	I miss having people around me^b^	170 (17.3)	278 (28.3)	536 (54.4)
10	I often feel rejected^b^	73 (7.5)	184 (18.7)	727 (73.8)
11	I can call on my friends whenever I need^a^	641 (65.2)	286 (29.1)	57 (5.7)

^a^ The positively formulated items (social loneliness),^b^ The negatively formulated items (emotional loneliness).

**Table 3:** Menopause Rating Scale domain deterioration according to loneliness perception

**Domains**	**Emotional Loneliness**		**Social Loneliness**		**General Loneliness**	
	**Yes** **433**	**No** **551**	**Yes** **415**	**No** **569**	**Yes** **438**	**No** **546**
Severe somatic deterioration	74 (17.0)	30 (5.4)	67 (16.1)	37 (6.5)	68 (15.5)	36 (6.5)
Severe psychological deterioration	182 (42.0)	87 (15.7)	160 (38.5)	109 (19.1)	177 (40.4)	92 (16.8)
Severe urogenital deterioration	157 (36.2)	89 (16.1)	144 (34.7)	102 (17.9)	158 (36.0)	88 (16.1)
Severe deterioration in quality of life	134 (30.9)	46 (8.3)	114 (27.4)	66 (11.6)	125 (28.5)	55 (10.0)

For all: *χ*^2^*P*<0.001. Data are presented as number (percent).

**Table 4:** Factors associated with loneliness perception among studied women Unadjusted logistic regression

**Variables**	**Emotional loneliness**		**Social loneliness**		**General loneliness**	
	**OR [95% CI]**	***P***	**OR [95% CI]**	***P***	**OR [95% CI]**	***P***
Severe somatic deterioration	3.57 [2.29-5.58]	<0.001	2.76 [1.81-4.22]	<0.001	2.60 [1.70-3.98]	<0.001
Severe psychological deterioration	3.86 [2.87-5.21]	<0.001	2.64 [1.98-3.53]	<0.001	3.34 [2.49-4.49]	<0.001
Severe urogenital deterioration	2.95 [2.18-3.98]	<0.001	2.43 [1.81-3.26]	<0.001	2.93 [2.17-3.96]	<0.001
Severe deterioration in quality of life	4.92 [3.41-7.08]	<0.001	2.88 [2.06-4.03]	<0.001	3.56 [2.51-5.04]	<0.001
High fear of COVID-19	1.97 [1.52-2.56]	<0.001	1.93 [1.49-2.51]	<0.001	1.97 [1.52-2.56]	<0.001
Dysfunctional levels of anxiety with COVID-19	3.57 [2.14-5.95]	<0.001	2.49 [1.54-4.02]	<0.001	2.69 [1.65-4.39]	<0.001
High religiosity	1.40 [1.07-1.81]	0.01	0.98 [0.76-1.28]	0.92	1.21 [0.93-1.57]	0.14

**Table 5:** Factors associated with loneliness perception among studied women Adjusted logistic regression

**Variables**	**Emotional loneliness**		**Social loneliness**		**General loneliness**	
	**OR [95% CI]**	***P***	**OR [95% CI]**	***P***	**OR [95% CI]**	***P***
Severe somatic deterioration	1.46 [0.88-2.43]	0.14	1.50 [0.92-2.42]	0.09	1.10 [0.67-1.81]	0.68
Severe psychological deterioration	2.52 [1.80-3.54]	<0.001	1.78 [1.27-2.48]	0.001	2.34 [1.67-3.29]	<0.001
Severe urogenital deterioration	1.90 [1.37-2.64]	<0.001	1.72 [1.25-2.37]	0.001	2.05 [1.48-2.84]	<0.001
High fear of COVID-19	1.39 [1.05-1.84]	0.02	1.54 [1.16-2.03]	0.002	1.48 [1.12-1.96]	0.006
Dysfunctional levels of anxiety with COVID-19	1.48 [0.84-2.62]	0.17	1.18 [0.69-2.02]	0.52	1.15 [0.67-2.00]	0.59
High religiosity	1.26 [0.95-1.66]	0.10	0.86 [0.65-1.13]	0.29	1.09 [0.83-1.44]	0.52

Variables included in the adjusted model: Severe somatic deterioration, Severe psychological deterioration, Severe urogenital deterioration, High fear of COVID-19, Dysfunctional levels of anxiety with COVID-19, High religiosity.
